# Finite element modeling of shape memory polyurethane foams for treatment of cerebral aneurysms

**DOI:** 10.1007/s10237-021-01540-7

**Published:** 2021-12-14

**Authors:** H. R. Jarrah, A. Zolfagharian, M. Bodaghi

**Affiliations:** 1 Department of Engineering, School of Science and Technology, Nottingham Trent University, Nottingham, NG11 8NS UK; 2 School of Engineering, Deakin University, Geelong, 3216 Australia

**Keywords:** Finite element modeling, SMPs, Shape memory foams, Cerebral aneurysms

## Abstract

In this paper, a thermo-mechanical analysis of shape memory polyurethane foams (SMPUFs) with aiding of a finite element model (FEM) for treating cerebral aneurysms (CAs) is introduced. Since the deformation of foam cells is extremely difficult to observe experimentally due to their small size, a structural cell-assembly model is established in this work via finite element modeling to examine all-level deformation details. Representative volume elements of random equilateral Kelvin open-cell microstructures are adopted for the cell foam. Also, a user-defined material subroutine (UMAT) is developed based on a thermo-visco-elastic constitutive model for SMPUFs, and implemented in the ABAQUS software package. The model is able to capture thermo-mechanical responses of SMPUFs for a full shape memory thermodynamic cycle. One of the latest treatments of CAs is filling the inside of aneurysms with SMPUFs. The developed FEM is conducted on patient-specific basilar aneurysms treated by SMPUFs. Three sizes of foams are selected for the filling inside of the aneurysm and then governing boundary conditions and loadings are applied to the foams. The results of the distribution of stress and displacement in the absence and presence of the foam are compared. Due to the absence of similar results in the specialized literature, this paper is likely to fill a gap in the state of the art of this problem and provide pertinent results that are instrumental in the design of SMPUFs for treating CAs.

## Introduction

A Cerebral Aneurysm (CA) is a weak or thin spot on an artery in the brain that balloons or bulges out and fills with blood. This phenomenon is common in 3–5% of people. A cerebral or intracranial aneurysm is an abnormal focal dilation of an artery in the brain that results from a weakening of the inner muscular layer (the intima) of a blood vessel wall. When this disease gets dangerous, there is the possibility of rupturing the arterial wall. This kind of hemorrhage can lead to a stroke, coma, or death (Brisman et al. 2006). Despite progress in surgical techniques and perioperative management, the rate of death because of rupturing in aneurysms is so high. Current treatment options are limited to invasive therapies (called microsurgical clipping) and endovascular coiling that both of them have high risk and side effects (Chalouhi et al. 2012; Ihn et al. 2018). Most CAs are small and 50–80% of them don’t rupture during the life span of patients. Thompson et al. (2019) expressed that the cause of creation of CAs can be local weakness and aneurysmal dilatation at the apex. Recently, He et al. (2021) presented a finite element implementation of arterial wall growth and remodeling with application to abdominal aortic aneurysms. The hemodynamically generated forces due to impingement of the central streams can be an important factor of degradation in the elastic membrane. Intracranial aneurysms are not considered sporicidal wounds although they have been described as a form of rare family (Chung et al. 2020).

**Fig. 1:**
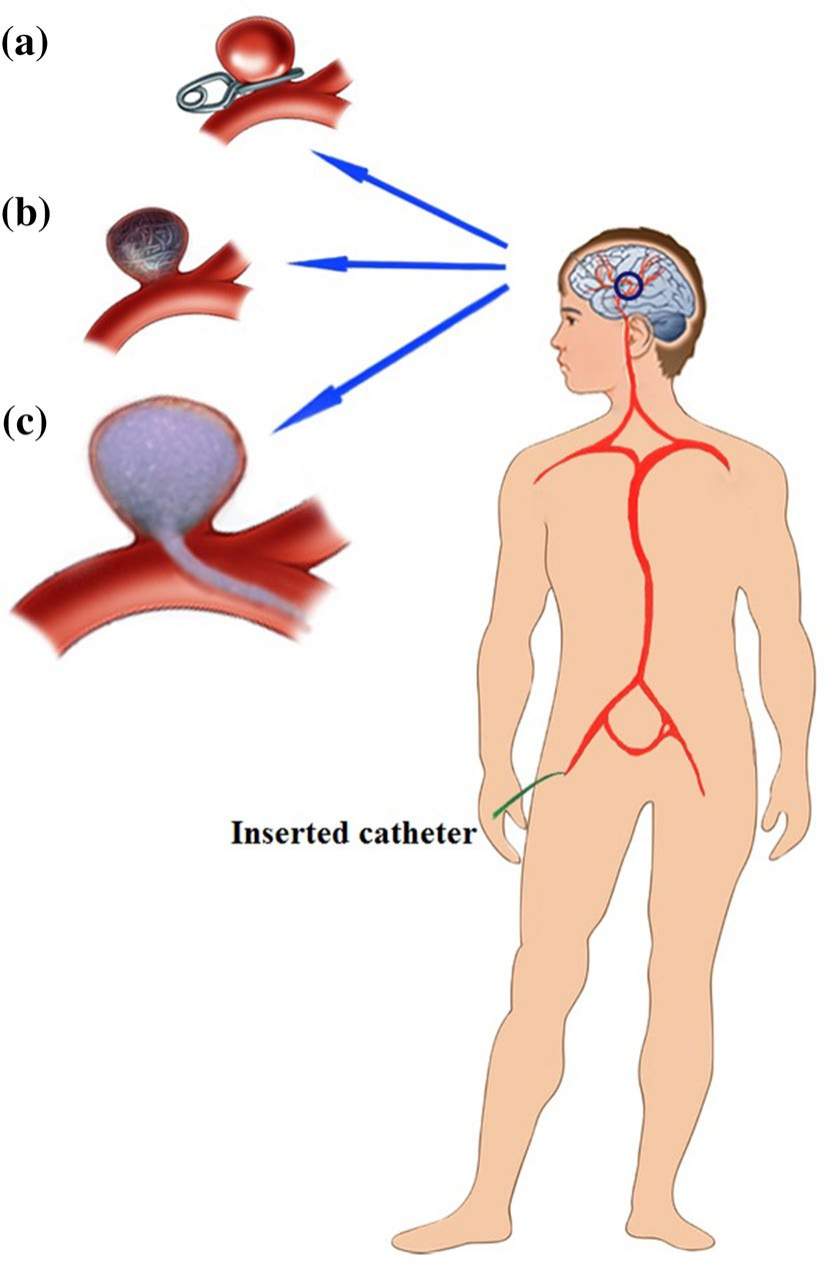
The variety of treatment for cerebral aneurysms: **a** clipping, **b** coiling, **c** foaming

**Fig. 2:**
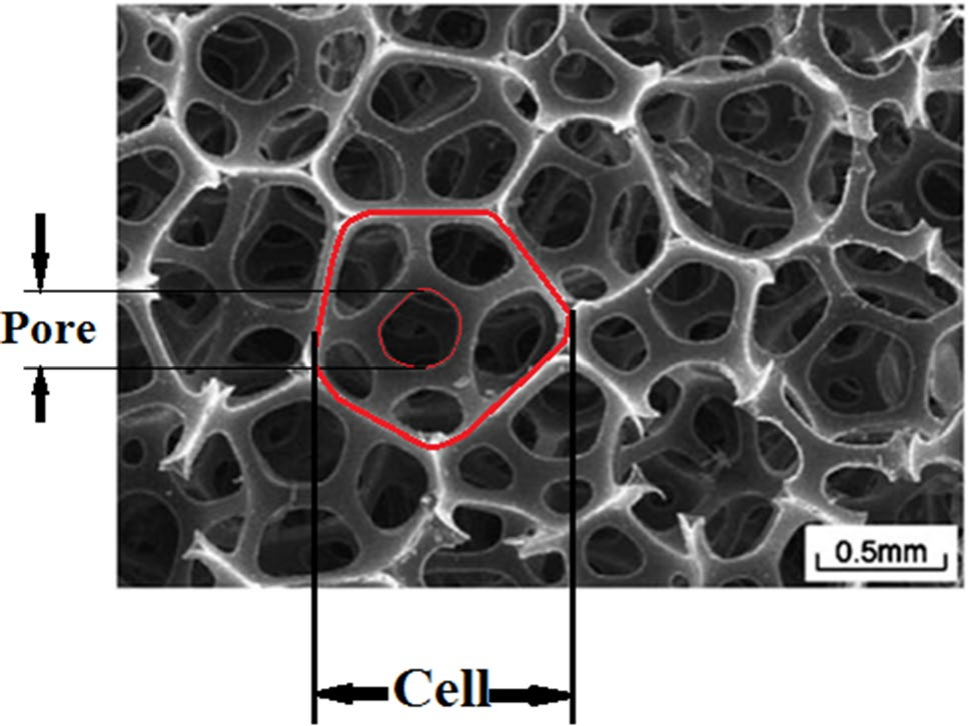
Micrograph showing cellular microstructure of the open-cell foam (Gong et al. 2005)

**Fig. 3:**
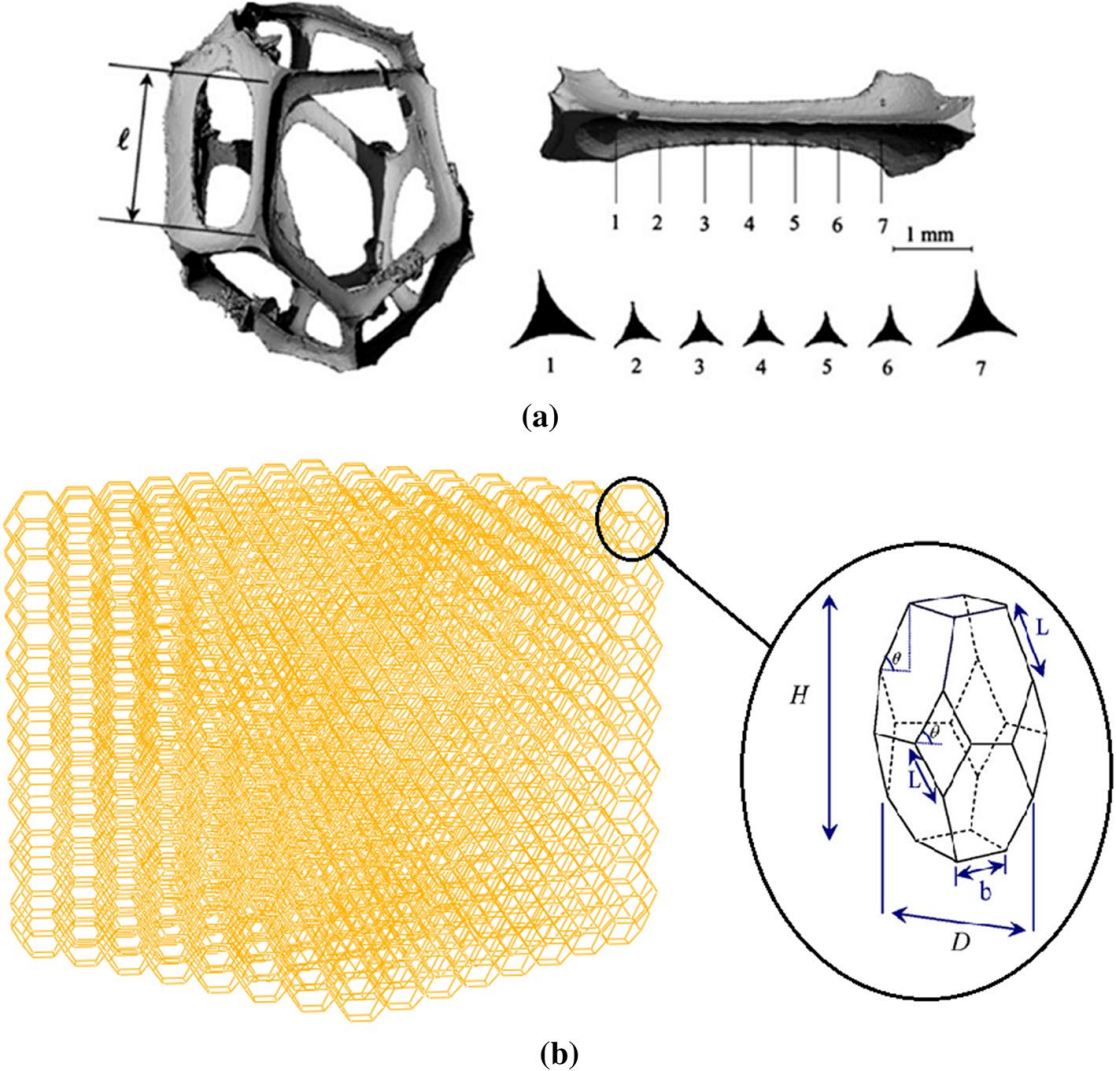
Geometric characteristics of the cells and equivalent Kelvin cell **a** microstructure of single cell (Demiray et al. 2009) and **b** micrograph showing of the beam

There are three treatments for this complication: (1) clipping (Fig. 1a), (2) coiling (Fig. 1b), (3) foaming (Fig. 1c).

Recently, Guglielmi Detachable Coil (GDC) has been more common for the treatment of aneurysms; of course, the type of remedy depends on aneurysm conditions. This method replaces clipping dramatically. In addition, this approach has been proved to be less invasive, leading to less brain injury (Le Roux and Winn 1998). In this procedure, an interventional neuro-radiologist with training in interventional radiology does endovascular coiling. During this method, general anesthesia is used (Qureshi et al. 2001). Although the percentage of success in clipping is higher than in coiling, coiling does not cause some problems, such as high risk, bleeding, hemorrhage, and incomplete occlusion (David et al. 1999). Surgeons can lead a coil inside of an aneurysm and try to locate the coil in the right position. Once the coil is inserted inside the aneurysm properly, a light electricity flow is applied to the coil. Although the GDC technique causes improvement in aneurysm treatment inside the skull significantly, this method has some drawbacks and it is not the best way for wide-necked aneurysms (Cloft et al. 2000). For instance, treatment of aneurysms with a neck dimension of less than 4 mm is probable between 57 and 85% with the GDC method, while for more than 4 mm it will reach 15–35% (Walsh et al. 1989; Zubillaga et al. 1994). In these cases, the coils move inside the parent artery and this movement increases the risk of aneurysm regrowth, rupture, and stroke. Incomplete filling is another disadvantage of the GDC technique (Willinsky 1999).

Nowadays, different types of bioparticles and smart materials are investigated with applications in surgery and medicine (SoltanRezaee and Bodaghi 2020). Shape memory polyurethane foams (SMPUFs) are being chosen as the best materials for filling and occluding the aneurysms (Ortega et al. 2013). Metcalfe et al. (2003) inserted shape memory polymer (SMP) foam inside of a dog’s aneurysm invasively. Researchers at the Lawrence Livermore National Laboratory (LLNL) presented a way of filling these materials because of the high deformation ability of foam proportional to temperature changes (Basheer et al. 2003). During this method, a piece of SMPUF is sent inside the aneurysm by catheter. Then a radially diffusing fiber located in the center of the foam will be responsible for heat transfer to the foam. This fiber is connected to a standard fiber optic cable that extends the whole length of the catheter. Energizing foam by diffusing fiber, the temperature of the foam will reach higher than glass transition temperature (${T}_{\mathrm{g}}$) and will be released foam and will occupy the aneurysm (Lendlein and Langer 2002). Recent studies in LLNL revealed that the rate of increasing foam volume is approximately 60 times (Small et al. 2007). SMPUFs have high applicability in hemostatic because of their high biocompatibility, high porosity, low density, and shape memory (Monroe et al. 2018). SMP foams have a much porosity space and when the temperature reaches more than ${T}_{\mathrm{g}}$, they will have the capability of compression into tiny volume (secondary shape). These foams have the ability to keep their shape in the secondary shape (De Nardo et al. 2009). This purpose is made possible by reaching temperature below the transition temperature, ${T}_{\mathrm{g}}$, via cooling. The transition temperature for filling the inside of an aneurysm should be in the range of 45–70 °C (Singhal et al. 2014). These types of foams were synthesized by Mitsubishi Heavy Industry (Tobushi et al. 2001a; b; Lendlein et al. 2010).

The literature survey reveals that most of research has been devoted to studying the possibility of using this type of material for treating aneurysms, while there is no study about the simulation of this process by finite element (FM) modeling. Also, it can be found that there is no comprehensive model for the simulation of SMP foams for a full shape memory cycle. Moreover, choosing the best size of SMPUF for filling the inside of CSs is another uninvestigated problem.

The structure of the foam cell is tiny and intricate, so observing its deformation is complicated. A cell assembly model is established via Finite Element (FE) modeling to examine all-level deformation details. Representative Volume Element (RVE) is the smallest volume over which a measurement can be made that will yield a value representative of the whole. In this paper, an RVE is established via FE modeling to simulate the geometry of the SMP foam. The properties of the model are defined according to the thermo-visco-elastic model. The FE modeling is carried out to predict both the stress–strain results and the shape memory cycle. Furthermore, the process of injection of SMPUFs inside the aneurysm is simulated and the distribution of stress and displacement for three sizes of SMPUF are compared in the cerebral aneurysm. Due to the absence of similar results in the specialized literature, it is expected that the results of this research would contribute to a better understanding of the application of SMPUFs and their performance for treating cerebral aneurysms.

## Modeling of SMP foams

### Geometry and properties of foam cells

The structure of foam is very complicated. Hence, the derivation of comprehensive governing equations that can introduce their behaviors properly is challenging. Gibson and Ashby (Gibson et al. 2010) presented a theory that connected the relative density of foam to its effective stiffness. Relative density is defined as the ratio of foam density $\left({\rho }^{*}\right)$ to density of those polymers $\left({\rho }_{\mathrm{s}}\right)$ that foam is made of. The same as this definition for density, it can be considered for the stiffness of foam. Gibson and Ashby (Gibson et al. 2010) assumed that cell foam is a cell cellular material. Also, they considered a cubic shape for every cell. Zhu et al. (1997) presented that the tetrakaidecahedron (the Kelvin cell) is the only polyhedron that can be assembled together to fill space. The microstructure of polyurethane (PU) foam was scanned by Gong et al. (2005), and the results illustrated that PU foam cells have 13.7 faces and 4.94 edges on each face. Figure 2 shows the micrograph of PU foam. An RVE of random equilateral Kelvin open-cell microstructures was used for modeling of foam. A tetrakaidecahedron cell has eight hexagonal, four-diamond, and two squares as shown in Fig. 3a. In this study, it is assumed that cells have an elongated tetrakaidecahedron. Zhu et al. (1997) derived governing equations for this cell. The required dimension of the cell is illustrated in Fig. 3b.

The governing equations on this tetrakaidecahedron are:

1 $${\raise0.7ex\hbox{$b$} \!\mathord{\left/ {\vphantom {b L}}\right.\kern-\nulldelimiterspace} \!\lower0.7ex\hbox{$L$}} = \sqrt 2 \cos \theta$$

2 $$H=4L\mathrm{sin}\theta$$

**Fig. 4:**
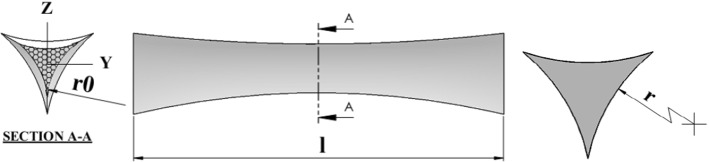
Definition of geometry of foam ligaments

The length of the ligaments (edges) is l. Ligaments don’t have a uniform cross-section ($A$) and they are different along the ligament. Gong et al. (2005) investigated different shapes for foam ligaments. This geometry is chosen as the best shape for the ligaments. He considered an equal cross-section and moments of inertia as detailed below. Figure 4 shows the dimensions of a ligament.

3 $$A=\left(\sqrt{3}-\frac{\pi }{2}\right){r}^{2}$$

4 $${I}_{y}={I}_{z}=\frac{1}{24}\left(20\sqrt{3}-11\pi \right){r}^{4}$$

As mentioned before, the relative density $(\gamma )$ is the ratio of the foam density $\left({\rho }^{*}\right)$ to the density of the solid material $\left({\rho }_{\mathrm{s}}\right)$. Thus:

5 $$\gamma =\frac{{\rho }^{*}}{{\rho }_{s}}=\frac{{V}_{s}}{{V}^{*}}=\frac{2A(2L+b)}{L\mathrm{sin}\theta {[2L\mathrm{cos}\theta +\sqrt{2}b]}^{2}}$$

Also, stiffness components can be written as:

6 $${E}_{x}=\frac{24{E}_{s}(T)I\mathrm{sin}\theta }{{L}^{2}[{cos}^{2}\theta +\frac{12I{sin}^{2}\theta }{A{L}^{2}}]{[2L\mathrm{cos}\theta +\sqrt{2}b]}^{2}}$$

7 $${E}_{y}={E}_{z}=\frac{12{E}_{s}I}{L\mathrm{sin}\theta [2{L}^{3}{sin}^{2}\theta +{b}^{3}+\frac{12I}{A}\left(2L{cos}^{2}\theta +b\right)]}$$

8 $${\nu }_{zx}={\nu }_{xz}=\frac{b\left(A{b}^{2}-12I\right)}{12I\left(2L{cos}^{2}\theta +b\right)+A(2{L}^{3}{sin}^{2}\theta +{b}^{3})}$$

9 $$\nu_{yx} = \nu_{xy} = \frac{{\left( {AL^{2} - 12I} \right)\left( {2L\cos \theta + \sqrt 2 b} \right)cos\theta }}{{2\left[ {12I\left( {2Lcos^{2} \theta + b} \right) + A\left( {2L^{3} sin^{2} \theta + b^{3} } \right)} \right]}}$$

**Fig. 5:**
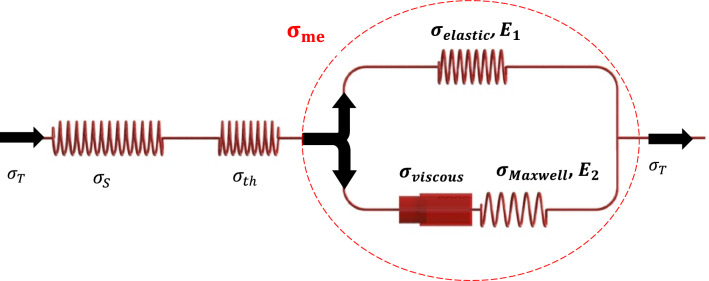
Three-dimensional rheological representation of viscoelastic model

Tobushi et al. (2001a; b) presented a rheological model (Fig. 5) for SMPs based on the thermo-visco-elastic model of polymers. Shape memory polymer foams have special specifications. The foam properties are completely different before and after the transition temperature. Also, the material has a viscous behavior and the material should have the ability to store the applied strain. The Tobushi model covers all these desired points and can fulfill them properly. Indeed, the mechanical part of rheological representation has two sections. One of them is the elastic part and another one is the viscous part. The elastic part is a spring and its stiffens express the elastic property of the foam. The viscous part is a dashpot damper that creates the viscous section of the foam. The dashpot damper coefficient expresses the viscous property of foam and changes with temperature. In this model, it is assumed that the total strain includes mechanical strain $\left({\varepsilon }_{\mathrm{me}}\right)$, thermal strain $\left({\varepsilon }_{\mathrm{th}}\right)$, and storage strain $\left({\varepsilon }_{\mathrm{S}}\right)$. Mechanical strain involves elastic strain $\left({\varepsilon }_{\mathrm{elastic}}\right)$ and viscous strain $\left({\varepsilon }_{\mathrm{viscous}}\right)$. Thus:

10 $${\varepsilon }_{\mathrm{T}}={\varepsilon }_{\mathrm{me}}+{\varepsilon }_{\mathrm{th}}+{\varepsilon }_{\mathrm{S}}$$

11 $${\varepsilon }_{\mathrm{me}}={\varepsilon }_{\mathrm{elastic}}+{\varepsilon }_{\mathrm{viscous}}$$

Also, the governing stresses of this rheological model are:

12 $${\sigma }_{\mathrm{T}}={\sigma }_{\mathrm{me}}={\sigma }_{\mathrm{th}}={\sigma }_{\mathrm{S}}$$

13 $${\sigma }_{\mathrm{me}}={\sigma }_{\mathrm{elastic}}+{\sigma }_{\mathrm{viscous}}={\sigma }_{\mathrm{elastic}}+{\sigma }_{\mathrm{maxwell}}$$

where ${\sigma }_{\mathrm{T}}$, ${\sigma }_{\mathrm{me}}$, ${\sigma }_{\mathrm{th}}$, ${\sigma }_{\mathrm{S}}$, ${\sigma }_{\mathrm{elastic}}$, ${\sigma }_{\mathrm{viscous},}$ and ${\sigma }_{\mathrm{maxwell}}$ are total stress and stress in mechanical parts, thermal stress, storage stress, elastic stress, viscous stress, and Maxwell stress, respectively.

A relation for stiffness (${E}_{\mathrm{s}}$), viscosity ($\mu$) and retardation time ($\lambda$) are defined in terms of temperature as:

$${E}_{s}\left(T\right)=\left(1-z\right){E}_{h}+z{E}_{l}$$

$$\mu \left(T\right)=\left(1-z\right){\mu }_{h}+z{\mu }_{l}$$

$$\lambda \left(T\right)=\left(1-z\right){\lambda }_{h}+z{\lambda }_{l}$$

where subscript of $h$ and $l$ are pointed to the properties below and above transition temperature, respectively. Furthermore, $z$ is a binding factor defined as:

14 $$z=1-{\left(1+{e}^{-a({T}_{g}-0.5{\Delta T}_{hl}-T)}\right)}^{-1}; a={\left|\mathrm{ln}\left(\frac{{\mu }_{h}}{{\mu }_{l}}\right)\right|}^{-1}$$

### UMAT subroutine

**Fig. 6:**
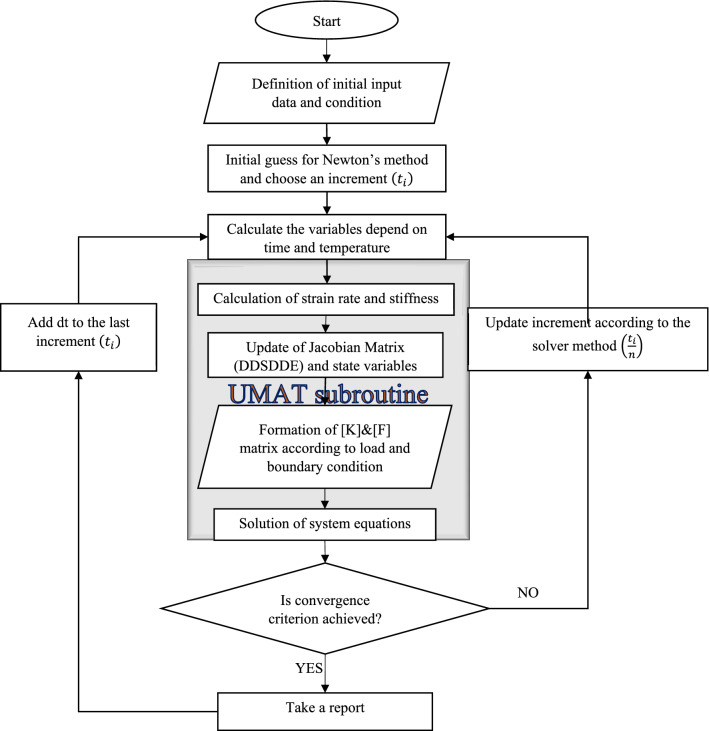
The computational algorithm for the solution of equations in ABAQUS

There are three general approaches for the simulation of SMP foams (Salman 2020). In the first one, which is called the macro-scale, the physical quantities are transferred from the micro-scale level to the macro-scale level. Recently, Jarrah et al. (2021) presented a VUMAT subroutine based on the constitutive model for the simulation of SMP foams. Additionally, Zolfagharian et al. (2021) introduced a simple approach for the simulation of shape memory polymers fabricated by 4D printing technology. They defined the properties proportional to temperature variations. The governing constitutive equations of the shape memory polymer foams are very complicated and need a VUMAT subroutine for defining their properties while this type of subroutine is heavy for running and it is not possible to use for many elements. Furthermore, the macro model is not able to simulate all the thermodynamic cycle. The other method for the simulation of SMP foams is the mesoscale model. According to this model, the materials have a cellular structure and the voids are distributed randomly inside the structures. In this study, the mesoscale model will be considered for simulating foams. Additionally, for the shape memory behavior, Tobushi model (2000) will be defined for each cell. Thus, in this study, a combination of mesoscale and Tobushi models is implemented for the simulation of SMP foams. A flowchart is introduced for this solution process, as illustrated in Fig. 6.

In this section, the mechanical constitutive behaviors of foams should be defined in ABAQUS software. In this subroutine, a material Jacobian matrix $\left(C=\frac{1}{J}\frac{\partial (J\sigma )}{\partial \Delta \varepsilon }\right)$ must be provided. Thus, the relation between stress and strain should be defined properly according to the viscoelastic model. Stress and strain are in the form of tensors depending on time and should be updated at the beginning of each increment. The relation between strains and stresses in this rheological model is expressed in Eq. (15).

15 $$\frac{{\sigma }_{T}}{\mu }+\frac{\dot{{\sigma }_{T}}}{\mu }-\frac{{\varepsilon }_{T}}{\lambda }+\dot{{\varepsilon }_{th}}+\dot{{\varepsilon }_{S}}=\dot{\varepsilon }$$

16 $$\left\{ \sigma \right\}_{{{\text{elastic}}}} = \left[ C \right]^{e} \left\{ \varepsilon \right\}_{e} ,\left\{ \sigma \right\}_{{{\text{Maxwell}}}} = \left[ C \right]^{M} \left\{ \varepsilon \right\}_{M} ,\left\{ \sigma \right\}_{{{\text{viscous}}}} = \left[ C \right]^{\mu } \left\{ {\dot{\varepsilon }} \right\}_{\mu }$$

17 $${\left[C\right]}^{-1}=\left[S\right]$$

where $\left[C\right]$ and $\left[S\right]$ are the stiffness tensor and compliance matrix, respectively.

18 $${\left\{\sigma \right\}}_{\mathrm{total}}={\left\{\sigma \right\}}_{\mathrm{Maxwell}}+{\left\{\sigma \right\}}_{\mathrm{Elastic}}$$

19 $${\left\{\sigma \right\}}_{\mathrm{Maxwell}}={\left\{\sigma \right\}}_{\mathrm{viscous}}$$

Like stress, the strain is written as:

20 $${\left\{\varepsilon \right\}}_{\mathrm{me}}={\left\{\varepsilon \right\}}_{\mathrm{Elastic}}={\left\{\varepsilon \right\}}_{\mathrm{Maxwell}}+{\left\{\varepsilon \right\}}_{\mathrm{viscous}}$$

Moreover, for time derivative, one has:

21a $${\left\{\dot{\varepsilon }\right\}}_{\mathrm{me}}={\left\{\dot{\varepsilon }\right\}}_{\mathrm{Elastic}}={\left\{\dot{\varepsilon }\right\}}_{\mathrm{Maxwell}}+{\left\{\dot{\varepsilon }\right\}}_{\mathrm{viscous}}$$

22b $$\left\{ \varepsilon \right\}_{i} = \left[ S \right]^{i} \left\{ \sigma \right\}_{i} \,{\text{and}}\,\left\{ \sigma \right\}_{i} = \left[ C \right]^{i} \left\{ \varepsilon \right\}_{i} \quad i\, = \,{\text{Maxwell}},\,{\text{elastic}},\,{\text{viscous}}$$

Thus:

23 $${\left\{\dot{\varepsilon }\right\}}_{\mathrm{me}}=\left(1+{{\left[C\right]}^{\mathrm{Maxwell}}}^{-1}+{\left[C\right]}^{E}\right)\dot{\varepsilon }+{{\left[C\right]}^{\mathrm{viscous}}}^{-1}{\left[C\right]}^{\mathrm{elastic}}\varepsilon$$

The corresponding Maxwell element Young’s modulus of the SMP is calculated as:

24 $${E}_{\mathrm{Maxwell}}={E}_{s}-\frac{3\mu }{\lambda }$$

In this study, the geometry is created according to the Kelvin cell that was explained in the earlier section. Beam element is used for the creation of the model. The type of this element is B33. This element is a cubic element that involves three additional variables. This element is a spatial element. The size of ligament section of the foam is negligible in compression with its length. Additionally, the run time would boom significantly if a solid element was chosen for this study. It is worth noting that the current finite element results follow experimental results very well.

**Fig. 7:**
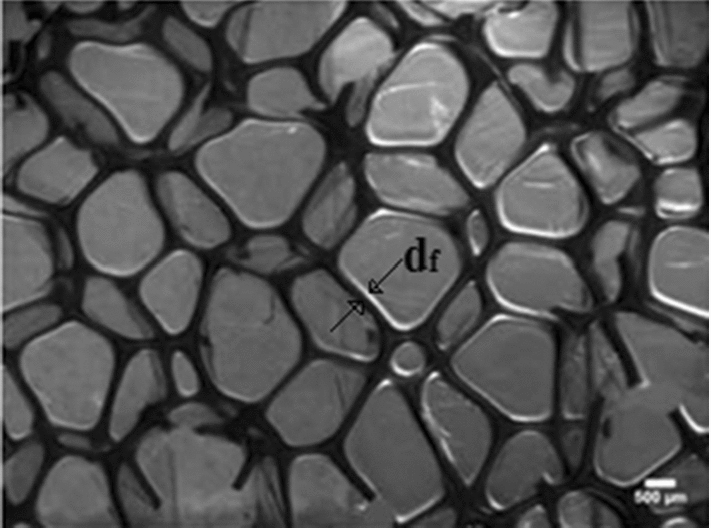
SEM micrographs of H20 polyuretfoam showing the cell structure and porosity of the foam ($d_{{\text{f}}} = 50\,\upmu {\text{m}}$, ppi = 60, scale bar 500 $\upmu {\text{m}}$) (Singhal et al. 2012)

There are different forms of SMP foams. In this study, a type of SMP foam should be chosen that has specific features. The first feature is transition temperature. For biomedical applications, the transition temperature of SMP foams should be near the body temperature. The second feature is that the SMP foam should have biocompatibility. Singhal et al. (Singhal, Rodriguez, et al. 2012) introduced different types of SMPUFs. They presented five kinds of SMPUFs known as H0, H20, H40, H60, and H80. They performed a biocompatibility test on them. The results of the tests are based on the level of cytokine production from the cells cultured on the foams and control substrates. This material was tested with an enzyme-linked immunosorbent assay and the result was acceptable for using this foam to treat for human body. The range size of the foam cell is limited. The foam properties are changed with the cell structure and raw ingredients. This size cell is the best choice because its stiffness is proportional to the aneurysm property. As an example, if the cell size is decreased, the stiffness will be increased, and this issue may lead to rupture in the aneurysm. There are different amounts for pores per inch (ppi). The pore size for the H20 is 419 μm The SEM micrographs of this foam are shown in Fig. 7. The requirement properties for the simulation in ABAQUS software are expressed in Table 1.

**Table 1:** Material and geometrical parameters required for simulation of PU SMP foams (De Nardo et al. 2009)$.$

$T_{{\text{g}}} \,\left( {^\circ {\text{K}}} \right)$	$\Delta T_{hl} \left( {^\circ {\text{K}}} \right)$	$E_{{\text{h}}} \,\left( {{\text{MPa}}} \right)$	$E_{{\text{l}}} \,\left( {{\text{MPa}}} \right)$	$\mu_{{\text{h}}} \left( {\text{GPa - s}} \right)$	$\mu_{{\text{l}}} \,\left( {\text{GPa - s}} \right)$	$\lambda_{{\text{h}}} \left( {\text{s}} \right)$	$\lambda_{{\text{l}}} \left( {\text{s}} \right)$
328	30	0.5	27	2.03	116	111	2480

$\theta \,\left( \circ \right)$	*r* (μm)	$H\,\left( {{\text{mm}}} \right)$	$L\,\left( {{\text{mm}}} \right)$	$b\,\left( {{\text{mm}}} \right)$	$D\,\left( {{\text{mm}}} \right)$	*l* (mm)	$\rho_{{{\text{neat}}}}$(neat polymer density)
65.9	25	0.44	0.58	0.331	20	126	1174

Pore cell size (μm)	$\alpha_{{\text{h}}} \, \left( {10^{ - 4} /^{ \circ } {\text{C}}} \right)$	$\alpha_{l} \, \left( {10^{ - 4} /^{ \circ } {\text{C}}} \right)$	*r* (μm)	$r_{0} \left( {\upmu {\text{m}}} \right)$	Cubic dimension	
					*a* (mm)	Height (mm)
					20	20
800	5.062	6.841	70	50		

### Verification of the model

The model is verified by the creation of a FEM model in ABAQUS software via the UMAT subroutine and mesco-scale for the simulation of SMPUFs. In this study, a shape memory thermodynamic cycle is applied to the SMPUF and FEM results are compared with experimental results presented in Singhal et al. (2012). There are four steps in the shape memory cycle.

1. Loading: the sample is heated up to a high temperature $({T}_{\mathrm{h}})$ and then a compressive strain is applied to it.
2. Fixing: while the constraints are on the sample, the temperature is decreased to a low temperature $({T}_{\mathrm{l}})$.
3. Heating: the sample is heated up to a high temperature $({T}_{\mathrm{h}})$ again.
4. Unloading: while the temperature is constant, the constraints are removed.

**Fig. 8:**
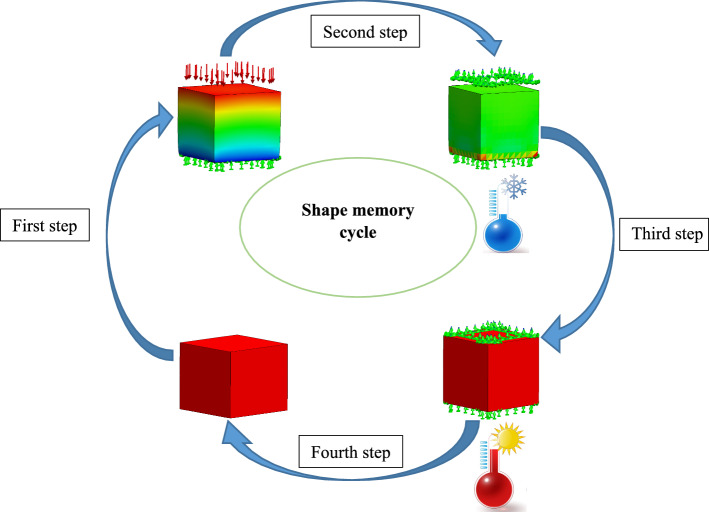
The shape memory cycle applied to the SMPUF

This cycle is schematically demonstrated in Fig. 8.

**Fig. 9:**
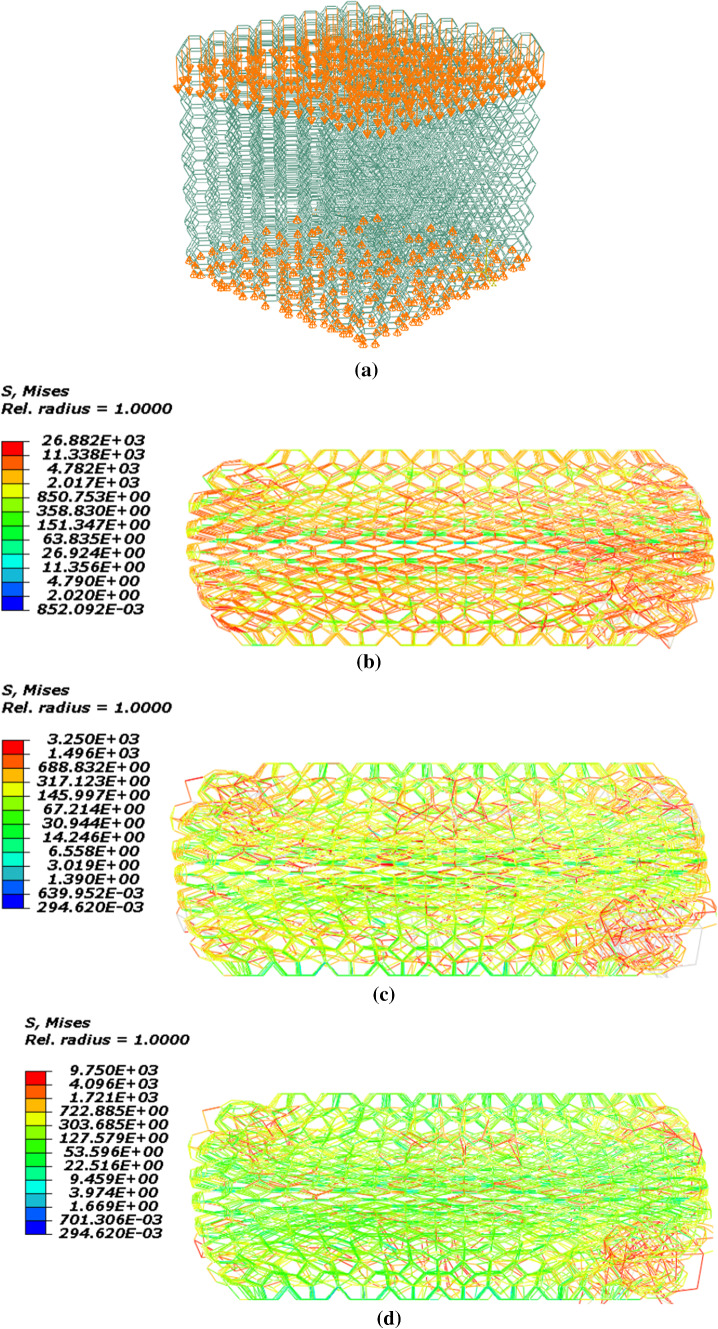

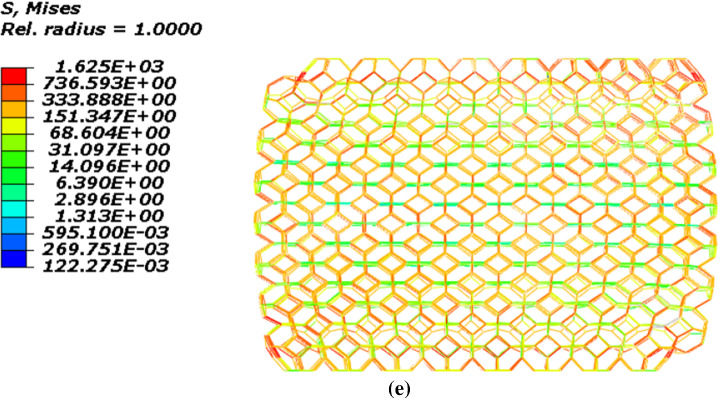
**a** Configuration of the foam model in ABAQUS, **b** stress contour of the foam at the end of the first step, **c** stress contour of the foam at the end of the second step, **d** stress contour of the foam at the end of the third step **e** stress contour of the foam at the end of the fourth step

**Fig. 10:**
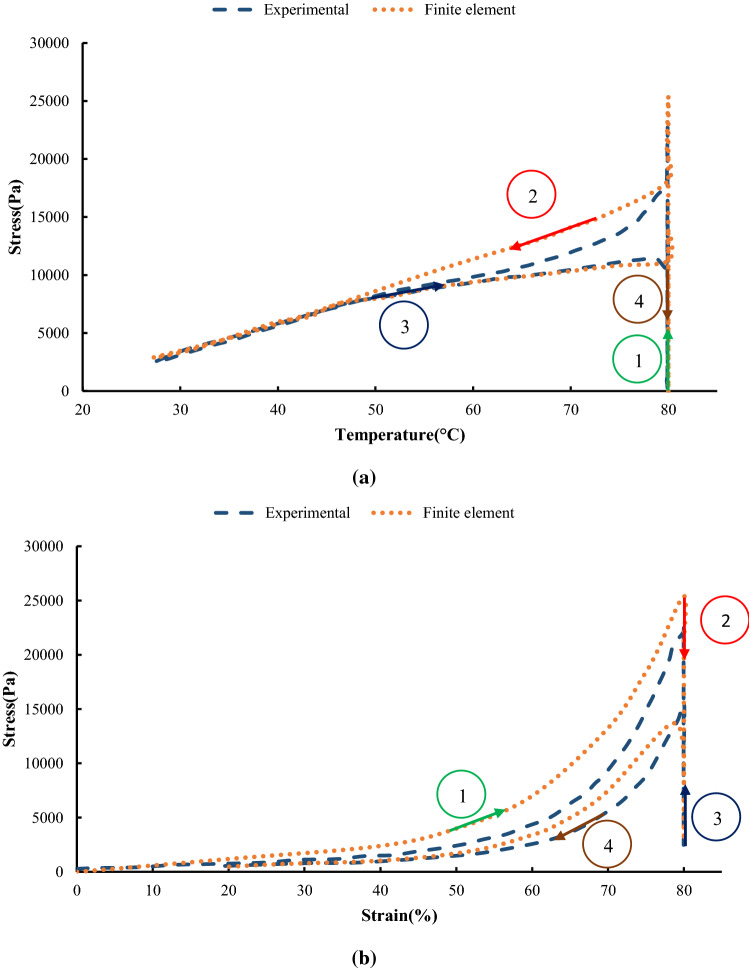
Comparison of experimental and FE results for the shape memory cycle: **a** stress versus temperature, **b** stress versus strain

The geometry of the model is created in the software according to the dimensions mentioned in Table 1. Furthermore, loading and boundary conditions are defined according to the test procedure. Also, the type of element in this model is the beam element that was mentioned before. This model is shown in Fig. 9a. Figure 9b–e demonstrates the stress contour of SMPUFs during the shape memory cycle. As seen in Fig. 10, the finite element results have suitable compatibility with the experimental results. Figure 10a illustrates the stress temperature governed by the model in the shape memory cycle. Also, Fig. 10b compares the stress–strain between experimental and FE results. In the first step, the temperature is constant at 80 °C. The relation between stress and strain is not linear and the material behaves like a hyper-elastic material. FE modeling predicts more stress rather than the experiment at the same strain. This difference is about 8%. In the second step, the foam is fixed at 80% strain. The stress is decreased to 2.5 kPa approximately. In the third step, the temperature will increase to 80 °C. In the last step, unloading is performed, and the strain of the foam reaches 0%.

There are some differences between experimental and finite element results due to different issues. For instance, in the theory, the temperature in the whole part of the foam was assumed to change at the same time while this hypothesis is so ideal and the temperature in different parts of the foam may not be the same. The other reason is that in the modeling the same size and shape were assumed for all cells of the foam while this may not be correct in reality.

## Geometry of artery and aneurysm

**Fig. 11:**
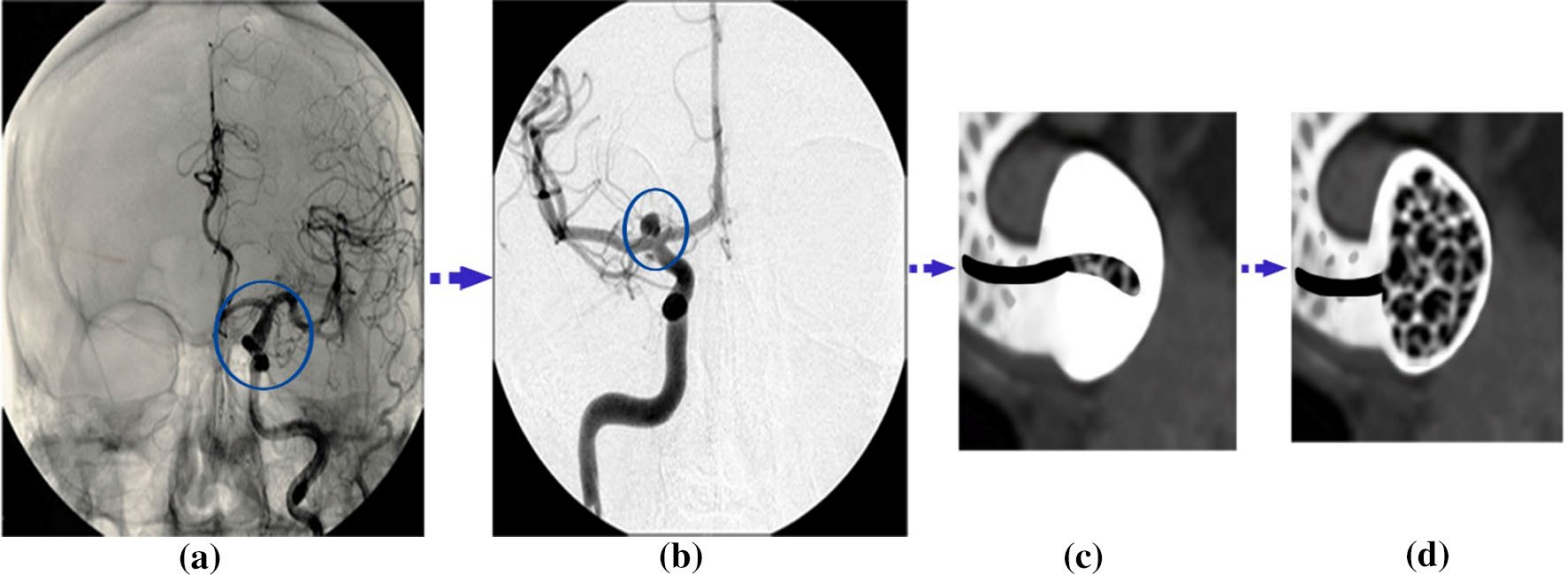
The process of treatment with SMPUFs: **a** reorganization of aneurysm with CT scan, **b** estimation of aneurysm size, **c** crimped (temporary shape) SMP foam device delivered to the aneurysm via catheter, **d** expansion of SMPUF within the aneurysm, as it recovers to its permanent shape via stimuli

In this section, the treatment of CAs is investigated by aiding SMPUFs. In this method, a SMPUF is inserted inside the aneurysm. Researchers at LLNL developed a technique to heat up actuated SMP foams inside aneurysms with optical energy (Ortega et al. 2007). First, a piece of SMPUF is moved inside aneurysms through a catheter. The foam is attached to a laser device that provides the required energy for heating the foam. Once the foam is in the right position, the laser applies energy to heat up the foam to a high temperature and the foam will be inflated. This process is shown schematically in Fig. 11c, b. As seen in Fig. 11a, the aneurysm is identified in the brain by CT scan technology. The size and exact dimension of aneurysm are determined in the next step (Fig. 11b). The appropriate size of the foam is inserted into the aneurysm by delivery catheter, see Fig. 11c and then it is expanded there as shown in Fig. 11d.

**Fig. 12:**
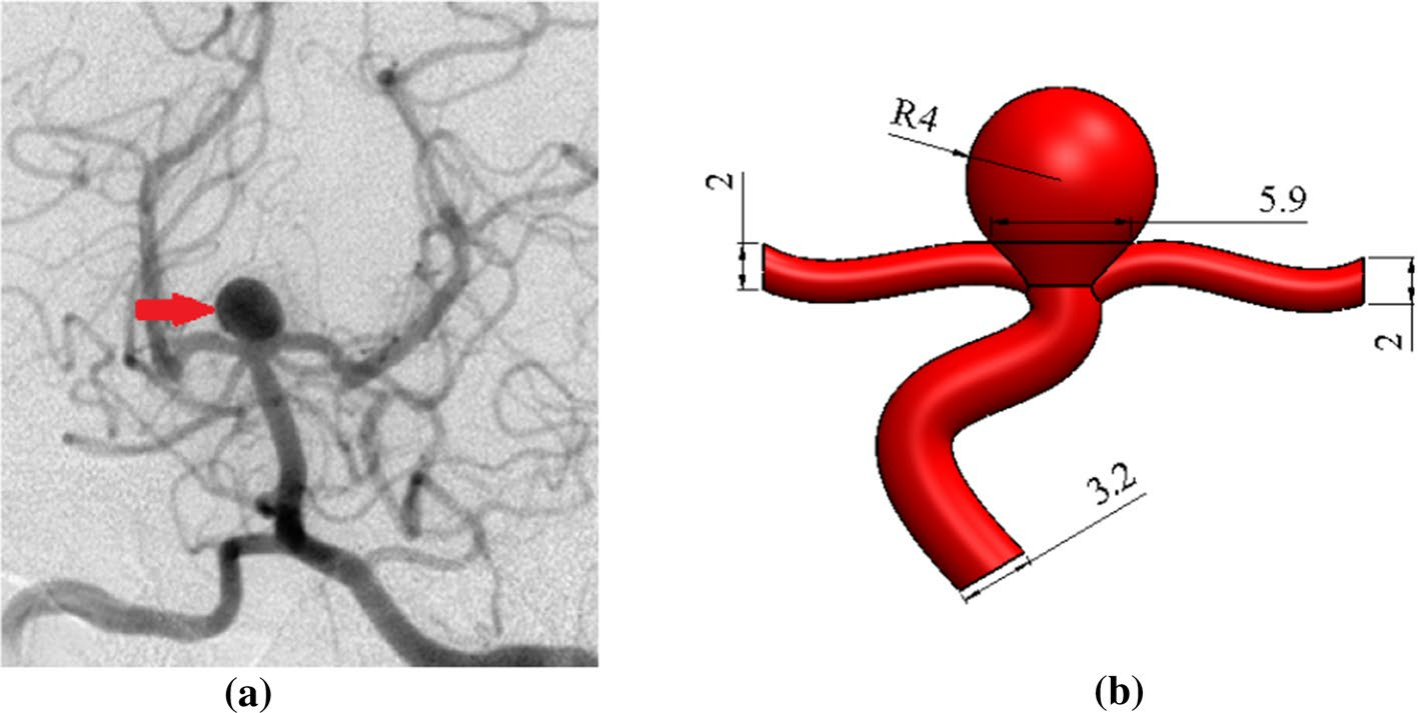
Demonstration of a berry aneurysm: **a** CT scan of the aneurysm (Florida State University 2010), **b** dimension of artery and aneurysm (in millimeter)

The inflated foam is prevented from circuiting blood inside of the aneurysm, so the applied pressure on the aneurysm wall declines. In this section, three sizes of SMPUFs are chosen for filling the inside of an aneurysm and the investigation of results. The dimensions of foam depend on the aneurysm dimensions. Generally, the geometry that governs this issue is complicated. For instance, the length of artery, thickness, and diameter of the aneurysm and artery, the exact shape of the aneurysm, and angle are so delicate. Therefore, some simplifications and approximations should be considered. Scott et al. (1972) measured the thickness of artery and aneurysm. The thickness of the artery is about 100–200 $\mathrm{\mu m}$ while for aneurysm it is 25 $\mathrm{\mu m}$ approximately. Scerrati et al. (2019) made a 3D anatomical model of aneurysm and artery by 3D printing for training and teaching. Ryan et al. (2016) proposed a 3D model of CA made by 3D printing technology as well. They used this model for training treatment of aneurysm by clipping method. Parlea et al. (1999) proposed geometry for aneurysm and its dimensions are illustrated in Fig. 12.

### Aneurysm and artery properties

**Fig.13:**
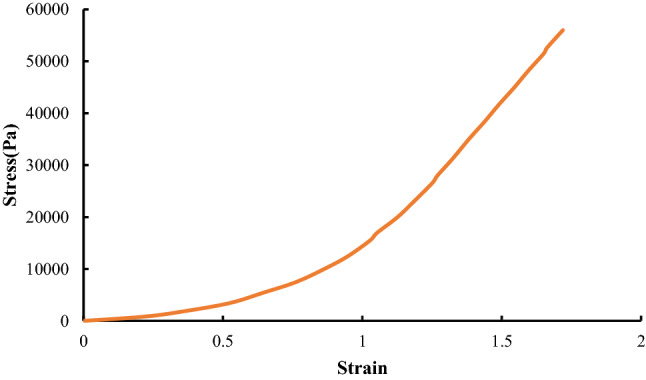
Stress–strain behavior of artery and aneurysm (Ferguson 1972)

The structural component of an aneurysm is collagen. Some studies reveal that the intra-aneurysmal pressure is the same as the systemic arterial pressure (Ferguson 1972). A study was performed to determine the elastic properties of the aneurysm wall (Scott et al. 1972). Analyses of the behavior of aneurysms revealed that the properties of aneurysm and cerebral arteries are different (Roach and Burton 1957). Wheatley et al. (2017) modeled intracranial aneurysms as thick-walled by non-linear elastic fiber-reinforced composites. Hassler (1961) and Nystrorn (1963) proved that aneurysms can be created until the internal elastic membrane get weak. Scott et al. (1972) conducted some experiments to compare the elastic properties of human intracranial arteries and aneurysms. They measured the stress–strain curves for both of them (Fig. 13). The wall thickness of the cerebral aneurysm was found to be 25  μm and average thickness of the aretey was determined as 175 μm. Harkness (1961) assumed that the aneurysm is made of collagen so the strength of wall is considered as the collagen strength. Thus, the strength of the aneurysm is set at 3 MPa. Also, Ley and Kim (2007) measured thermophysical parameters of the artery. Additionally, Hwang et al. (2012) fabricated an aneurysm model by latex for investigation of the effects of expansion, and the strength was same as aneurysm. Table 2 shows these quantities.

**Table 2:** Thermo-mechanical properties of the artery

Thermal conductivity $\left( {{\text{W/m}}.^\circ {\text{K}}} \right)$	Density $\left( {{\text{Kg/m}}^{3} } \right)$	Specific heat capacity $\left( {{\text{J/Kg}}.^\circ {\text{K}}} \right)$	Strength $\left( {{\text{MPa}}} \right)$
0.476	1075	3490	3

**Fig. 14:**
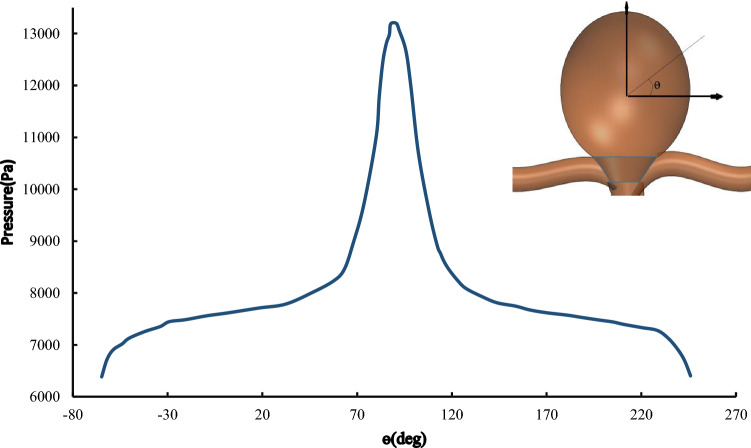
Pressure distribution across the aneurysm wall (Ortega et al. 2007)

Dorfmann et al. (2010) measured distribution of the wall pressure of patient-based lumen cast at a series of physiologically relevant steady flow rates. Ortega et al. (2007) conducted a Computational Fluid Dynamics (CFD) model for the estimation of pressure near the aneurysm. They extracted the pressure around the aneurysms according to the size of aneurysms as demonstrated in Fig. 14. The arteries have hyper-elastic behaviors. Various hyper-elastic models are regularly used to represent the large deformation behaviors of these materials (Dorfmann et al. 2010). In this study, Neo-Hookian hyper-elastic model is selected that has shown good accuracy for simulating deformations within the range investigated here. The C3D4H is used in this study. This element is a solid element with 4 nodes in the corners and the shape is linear tetrahedron. This element is used for hyper-elastic materials with a linear pressure. The solver links temperature and displacement. Additionally, the responses are in a steady-state manner. There is an interaction between foam and aneurysm. The type of this interaction is assumed contact with an interaction between node and surface. The pressure is applied to the foam according to Fig. 14. The relation between stress and strain in this hyperelastic material is demonstrated in Fig. 13. Meanwhile, the required boundary conditions for this simulation should be considered. The entrance and output of the artery are considered axisymmetric constrained.

## Numerical results and discussion

**Fig. 15:**
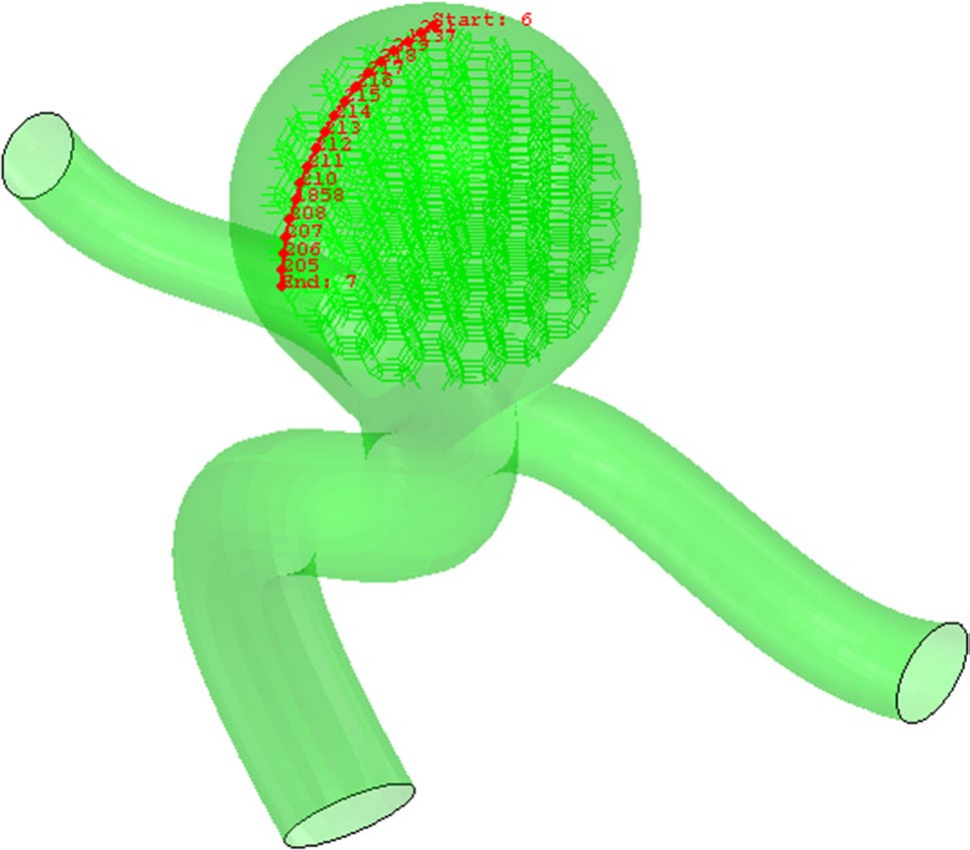
The chosen path for measurement data

**Fig. 16:**
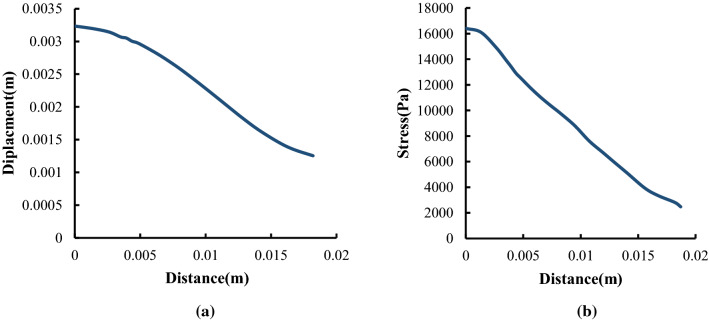
Distribution of **a** displacement and **b** stress on the meridian of aneurysm

**Fig. 17:**
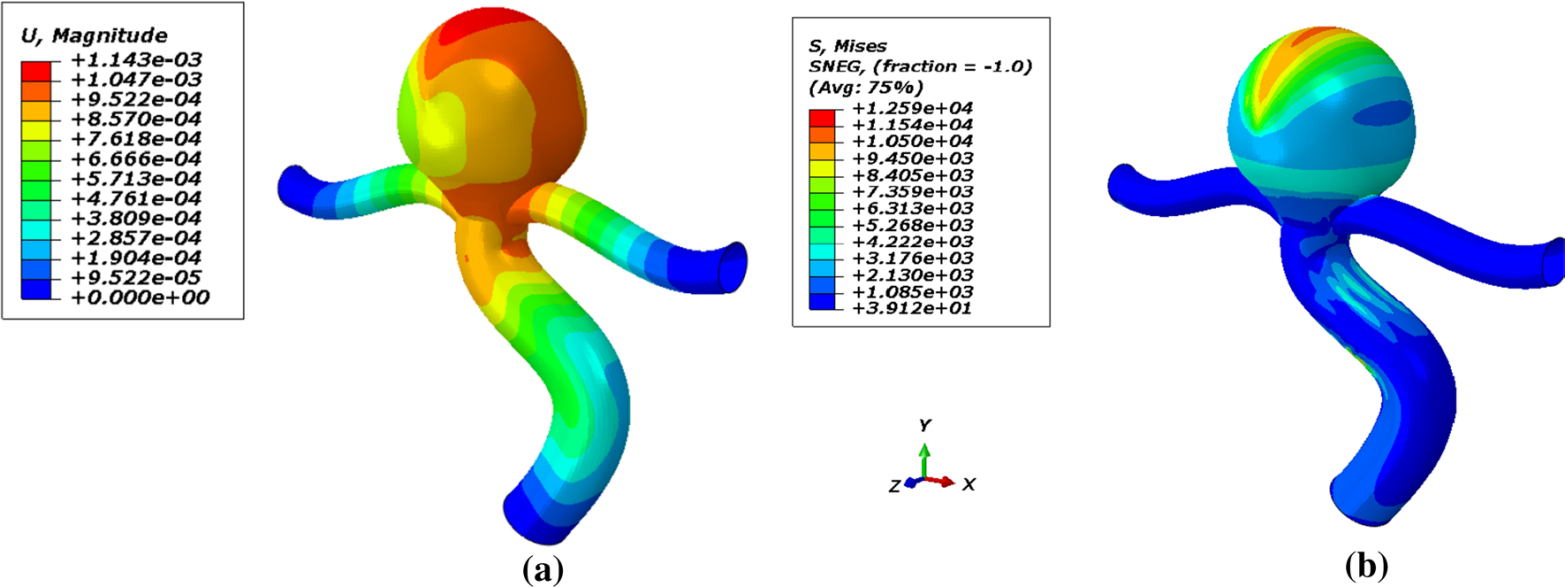
The contour of **a** displacement and **b** stress in the absence of the SMP foam

In this section, first of all, the artery with an aneurysm as shown in Fig. 12 is imported into ABAQUS, then the material properties (Fig. 13) are assigned to this geometry. Ortega et al. (2007) calculated the pressure inside of aneurysms with help of CFD. There is an interaction between the foam and the aneurysm. This type of interaction is frictionless and the pressure on this foam will cause stress on the aneurysm. Also, Fig. 14 shows the distribution of the blood pressure on the foam. This pressure is variable, and changes according to the location of foam. Furthermore, the boundary condition on three free sections of the artery is restricted. The effect of stress on the aneurysm wall will be investigated in this step. First, the effect of pressure is considered on the wall aneurysm in the absence of SMP foam, then the SMP foam is inserted inside the aneurysm and the effect of pressure on the wall is studied in the presence of SMP foam. A path (Fig. 15) was chosen for investigating the results. This path is located on the meridian of the aneurysm. Also, the distribution of the stress in this path has the most variations. Figure 16 shows the distribution of displacement and stress on the meridian of the aneurysm. As can be seen in this figure, the displacement and stress in the pick of the aneurysm is the maximum amount. In this situation, there is the highest amount of pressure distribution in the aneurysm. Thus, the peak of stress and displacement happens there. Figure 17 demonstrates the stress and displacement contour in the software for the aneurysm in the absence of SMP foam.

**Fig. 18:**
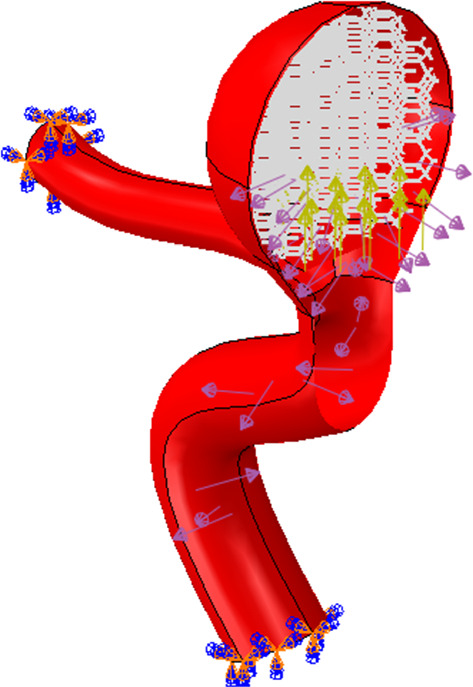
The applied boundary conditions and load on the model

**Fig. 19:**
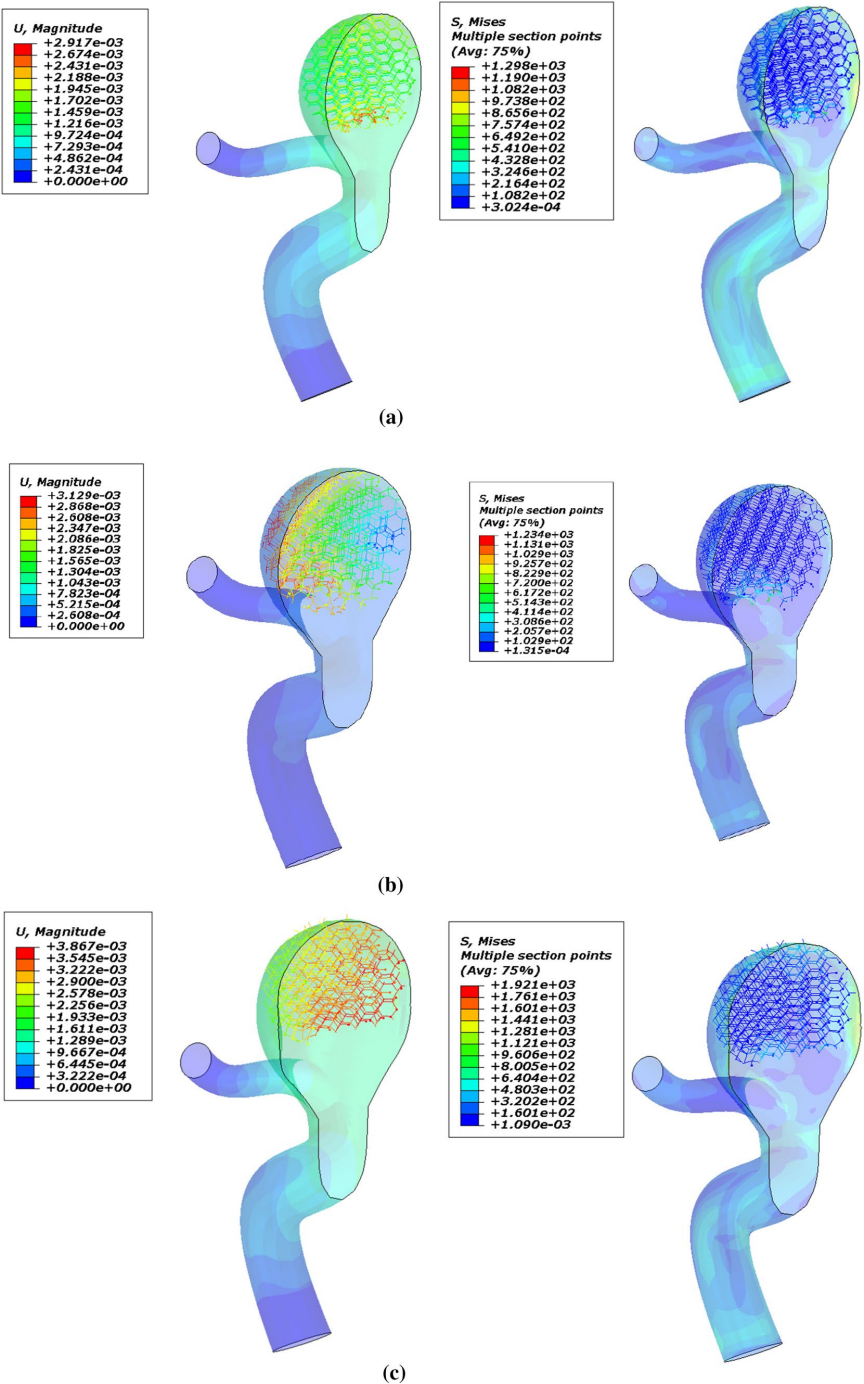
The applied boundary conditions and load on the model. **a** 7.5 mm, **b** 7 mm, **c** 6.5 mm

**Fig. 20:**
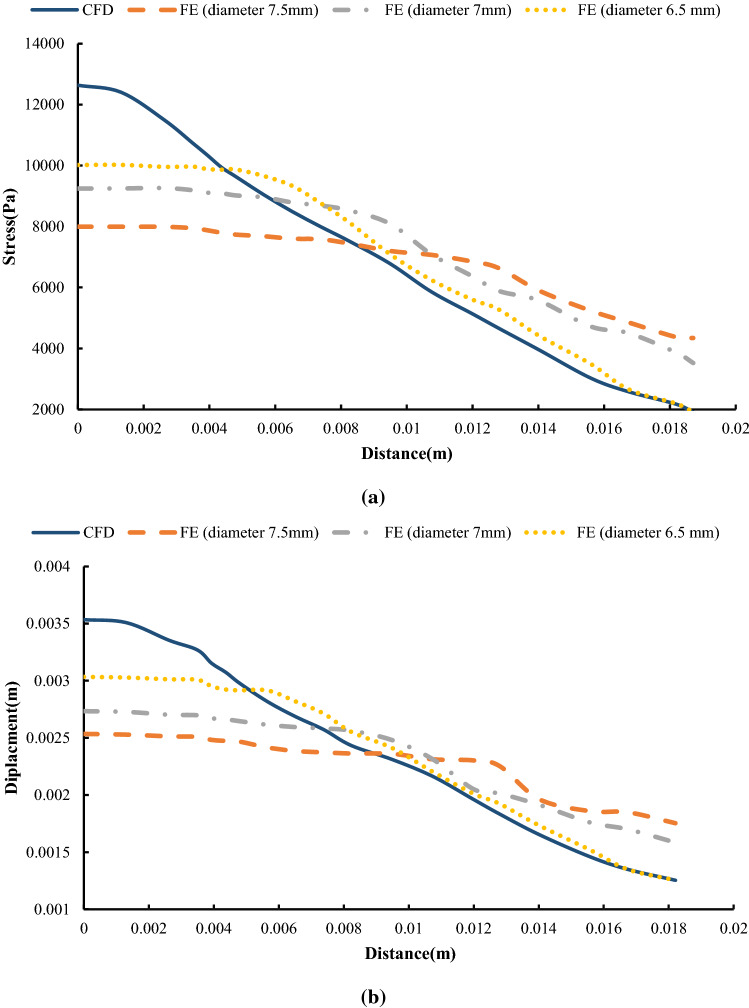
Distribution of **a** stress and **b** displacement in the chosen path and comparison with experimental results

In the following, the SMPUF in three sizes is inserted inside the aneurysm. The size of the foams is smaller than the aneurysm and its diameter is 7.5, 7, and 6.5 mm. The size range of the foam for choosing this treatment is limited. For instance, it is not possible to choose bigger, because it would cause occlusion in arteries and maybe itself causes a stroke. On the other hand, the smaller size also is not possible. In this manner, the foam might get off from aneurysms and it would cause an occlusion again. Additionally, once the size of foam is small, the blood will circulate around the foam and it ruptures the aneurysm. There are two steps in this simulation. The first step: applying heat to foam that increases its size. The second step: applying pressure to the foam that is the cause of blood pressure. Figure 18 shows the applied boundary conditions and loads on the foam and aneurysm. Also, there is an interaction between foam and aneurysm that is a type of frictionless contact. This interaction is chosen because of different reasons. Firstly, there is no movement or sliding between the aneurysm and the foam so defining friction between them is not very significant. Secondly, defining friction would boom the run time and the convergence would be decreased by this option. Finally, the contact area between the aneurysm and the foam is so low that can be considered negligible. The temperature of SMP foam is increased until the temperature reaches a high temperature. In this manner, the pressure is applied to the foam and then the stress and displacement distribution of stress on the aneurysm wall is investigated. The stress and displacement contours of these foams are shown in Fig. 19. The size of foam in Fig. 19a is 7.5 mm. When the temperature is applied to the SMPUF, the size of the foam will be increased, and this inflation is caused to create stress between the aneurysm wall and the foam. Then the blood pressure is applied to the foam. First, the foam is compressed and then the applied pressure on the aneurysm wall is decreased. The size of foam in Fig. 19b is 7 mm. This foam is smaller and when the size of the foam is boosted and the blood pressure is applied to it, the SMPUF is compressed more than when the diameter of foam is 7.5 mm. The size of foam in Fig. 19c is 6.5 mm. This size of the foam is compressed more than other sizes. The distribution of stress and displacement in the chosen path (Fig. 15) is demonstrated in Fig. 20 and the results are compared with the CFD results when there is no foam inside of the aneurysm. As can be seen in this figure, when the foam is inserted inside of the aneurysm, the highest amount of stress is declined. For the size of 7.5 mm, the maximum amount of stress is 8 kPa, while for the sizes of 7 and 6.5 mm it is 9 and 10 kPa respectively. Also, the highest amount of displacement is decreased when the foams fill inside of aneurysm. The maximum displacement for the foam with size of 7.5 mm is 2.5 mm, while this amount for the sizes of 7 and 6.5 mm is 2.7 and 3 mm respectively. Additionally, the distribution of stress and displacement has become more uniform. When the size of foam is bigger, the uniform distribution becomes longer.

## Conclusion

A study on the thermo-mechanical behavior of shape memory polymer foams was presented. The main features of SMP foams under the shape memory cycle were captured based on the thermo-visco-elastic constitutive model. Also, the geometry of the foam was defined based on the cell-assembly model. RVEs of random equilateral Kelvin open-cell microstructures were modeled for the open-cell foam. A UMAT subroutine was developed for defining the shape memory behavior. The model was able to capture the thermo-mechanical response of SMP foams for a shape memory cycle. In the last section, a SMPUF was used for filling inside an aneurysm. In this type of treatment, the SMPUF prevents the circuit of blood inside the aneurysm and causes a decrease in the applied stress on the walls. Furthermore, the distribution of stress on the aneurysm wall was found to be more uniformed. Thus, this study has presented a new method for choosing the best size of SMPUFs for better treatment of cerebral aneurysms with the aid of finite element method. According to the size of the aneurysm, the best size of foam could be chosen for treating it. The aneurysms have different sizes. This approach is more applicable for a small aneurysm's size. This paper presents the idea that the size of the foam should be selected according to the size of the aneurysm. It means that after a CT scan of the aneurysm, the model will be imported into the ABAQUS software and our model will evaluate the best size of the foam for treatment. Furthermore, according to the strength of an aneurysm, the model will be able to be used for a feasibility study for the treatment of the aneurysm. Also, with the aid of this approach, all processes of treatment can be simulated in the finite element software. Due to lack of any numerical data on shape memory foams for cerebral aneurysms, it is expected that the results supplied in the present work will be instrumental toward a reliable design of SMPUFs for treating cerebral aneurysms.

## References

[CR1] Basheer C (2003). Determination of organic micropollutants in rainwater using hollow fiber membrane/liquid-phase microextraction combined with gas chromatography–mass spectrometry. J Chromatogr A.

[CR2] Brisman JL (2006). Cerebral aneurysms. N Engl J Med.

[CR3] Chalouhi N (2012). Biology of intracranial aneurysms: role of inflammation. J Cereb Blood Flow Metab.

[CR4] Chung J (2020). Scalp thickness as a predictor of wound complications after cerebral revascularization using the superficial temporal artery: a risk factor analysis. Acta Neurochir.

[CR5] Cloft HJ (2000). Use of three-dimensional Guglielmi detachable coils in the treatment of wide-necked cerebral aneurysms. Am J Neuroradiol.

[CR6] David CA (1999). Late angiographic follow-up review of surgically treated aneurysms. J Neurosurg.

[CR7] De Nardo L (2009). Shape memory polymer foams for cerebral aneurysm reparation: effects of plasma sterilization on physical properties and cytocompatibility. Acta Biomater.

[CR8] Demiray S (2009). Investigation of the fatigue behavior of open cell foams by a micromechanical 3-D model. Mater Sci Eng A.

[CR9] Dorfmann A (2010). Evaluating patient-specific abdominal aortic aneurysm wall stress based on flow-induced loading. Biomech Model Mechanobiol.

[CR10] Ferguson GG (1972). Physical factors in the initiation, growth, and rupture of human intracranial saccular aneurysms. J Neurosurg.

[CR11] Florida State University College of Medicine (2010) https://www.tau.ac.il/medicine/tau-only/webpath/radiol/cnsrad/brain121.htm. The Internet Pathology Laboratory for Medical Education

[CR12] Gibson LJ (2010). Cellular materials in nature and medicine.

[CR13] Gong L (2005). Compressive response of open-cell foams. Part i: morphology and elastic properties. Int J Solids Struct.

[CR14] Harkness R (1961). Biological functions of collagen. Biol Rev.

[CR15] Hassler O (1961). Morphological studies on the large cerebral arteries, with reference to the aetiology of subarachnoid hemorrhage. Acta Psychiatr Scand.

[CR16] He X (2021). Estimating aortic thoracic aneurysm rupture risk using tension–strain data in physiological pressure range: an in vitro study. Biomech Model Mechanobiol.

[CR17] Hwang W (2012). Estimation of aneurysm wall stresses created by treatment with a shape memory polymer foam device. Biomech Model Mechanobiol.

[CR18] Ihn YK (2018). Complications of endovascular treatment for intracranial aneurysms: management and prevention. Interval Neuroradiol.

[CR19] Jarrah HR (2021). Nonlinear finite element modelling of thermo-visco-plastic styrene and polyurethane shape memory polymer foams.

[CR20] Le Roux PD, Winn HR (1998). Management of the ruptured aneurysm. Neurosurg Clin N Am.

[CR21] Lendlein A, Langer R (2002). Biodegradable, elastic shape-memory polymers for potential biomedical applications. Science.

[CR22] Lendlein A (2010). Shape-memory polymers as a technology platform for biomedical applications. Expert Rev Med Devices.

[CR23] Ley O, Kim T (2007). Calculation of arterial wall temperature in atherosclerotic arteries: effect of pulsatile flow, arterial geometry, and plaque structure. Biomed Eng Online.

[CR24] Metcalfe A (2003). Cold hibernated elastic memory foams for endovascular interventions. Biomaterials.

[CR25] Monroe MBB (2018). Multifunctional shape-memory polymer foams with bio-inspired antimicrobials. ChemPhysChem.

[CR26] Nyström S (1963). Development of intracranial aneurysms as revealed by electron microscopy. J Neurosurg.

[CR27] Ortega J (2007). Vascular dynamics of a shape memory polymer foam aneurysm treatment technique. Ann Biomed Eng.

[CR28] Ortega J (2013). Virtual treatment of basilar aneurysms using shape memory polymer foam. Ann Biomed Eng.

[CR29] Parlea L (1999). An analysis of the geometry of saccular intracranial aneurysms. Am J Neuroradiol.

[CR30] Qureshi AI (2001). Endovascular treatment of intracranial aneurysms by using Guglielmi detachable coils in awake patients: safety and feasibility. J Neurosurg.

[CR31] Roach MR, Burton AC (1957). The reason for the shape of the distensibility curves of arteries. Can J Biochem Physiol.

[CR32] Ryan JR (2016). Cerebral aneurysm clipping surgery simulation using patient-specific 3D printing and silicone casting. World Neurosurg.

[CR33] Salman MA (2020) Multiscale modelling of nano-clay filled shape memory polymer foams, Dissertation, xxii, 162

[CR34] Scerrati A (2019). A workflow to generate physical 3D models of cerebral aneurysms applying open source freeware for CAD modeling and 3D printing. Interdiscip Neurosurg.

[CR35] Scott S (1972). Comparison of the elastic properties of human intracranial arteries and aneurysms. Can J Physiol Pharmacol.

[CR36] Singhal P (2012). Ultra low density and highly crosslinked biocompatible shape memory polyurethane foams. J Polym Sci Part B Polym Phys.

[CR37] Singhal P (2014). Low density biodegradable shape memory polyurethane foams for embolic biomedical applications. Acta Biomater.

[CR38] Small W (2007). Shape memory polymer stent with expandable foam: a new concept for endovascular embolization of fusiform aneurysms. IEEE Trans Biomed Eng.

[CR39] SoltanRezaee M, Bodaghi M (2020). Simulation of an electrically actuated cantilever as a novel biosensor. Sci Rep.

[CR40] Thompson JW (2019). In vivo cerebral aneurysm models. Neurosurg Focus.

[CR41] Tobushi H (2000). Thermomechanical constitutive modeling of polyurethane-series shape memory polymer. Mater Sci Forum.

[CR42] Tobushi H (2001). Thermomechanical properties of polyurethane-shape memory polymer foam. J Intell Mater Syst Struct.

[CR43] Tobushi H (2001). Thermomechanical constitutive model of shape memory polymer. Mech Mater.

[CR44] Walsh JT (1989). Er: YAG laser ablation of tissue: effect of pulse duration and tissue type on thermal damage. Lasers Surg Med.

[CR45] Wheatley BB (2017). A validated model of passive skeletal muscle to predict force and intramuscular pressure. Biomech Model Mechanobiol.

[CR46] Willinsky RA (1999). Detachable coils to treat intracranial aneurysms. CMAJ.

[CR47] Zhu H (1997). Analysis of the elastic properties of open-cell foams with tetrakaidecahedral cells. J Mech Phys Solids.

[CR48] Zolfagharian A (2021). 4D printing classroom in modern interactive learning environments. Bioprinting.

[CR49] Zubillaga AF (1994). Endovascular occlusion of intracranial aneurysms with electrically detachable coils: correlation of aneurysm neck size and treatment results. Am J Neuroradiol.

